# Computed Tomography and a Dental Intraoral Scanner to Generate Three-Dimensional Models of the Beaks of Three Bird Species

**DOI:** 10.3390/vetsci12040331

**Published:** 2025-04-03

**Authors:** Gabriel Corrêa de Camargo, Sheila Canevese Rahal, Reinaldo Abdala Junior, Jeana Pereira da Silva, Daniel Simões da Silva, Maria Cristina Reis Castiglioni, Ricardo Shoiti Ichikawa, Bruno Critelli Carvalho

**Affiliations:** 1 Department of Veterinary Surgery and Animal Reproduction, School of Veterinary Medicine and Animal Science, São Paulo State University (UNESP), Botucatu 18618-681, Brazil; gabriel.c.camargo@unesp.br (G.C.d.C.); jeana.silva@unesp.br (J.P.d.S.); daniel.simoes@unesp.br (D.S.d.S.); m.castiglioni@unesp.br (M.C.R.C.); ricardo.ichikawa@unesp.br (R.S.I.); 2 UniFSP (Centro Universitário Sudoeste Paulista), Odontologia, Campus Avaré, Avaré 18707-150, Brazil; cdpiraju2@hotmail.com; 3 Prime Center of Veterinary Diagnostic, Sorocaba 18020-003, Brazil; brunoneurovet@gmail.com

**Keywords:** neotropical species, avian bills, measurements, imaging

## Abstract

This study aimed to assess the beaks of Neotropical birds using two scanning techniques—CT and a dental intraoral scanner—along with macroscopic analysis. Six specimens per family were selected, including parakeets, red-legged seriemas, and black vultures. The upper beaks were measured in the CT sagittal view for length along the longitudinal axis and height on the transverse axis. The same measurements were performed on the 3D images. Additionally, beak width in the middle of the *naris* area, as well as the length and height of the *nares*, were measured on the 3D images. The closed polygon tool outlined the beak, generating volume in cm^2^. The 3D images obtained with the dental scanner were measured, similarly to those from 3D-CT scans for the beaks’ length, height, and width. Macroscopic measurements of the beaks were also conducted. Some differences in beak measurements between imaging methods were verified. In conclusion, both techniques are effective, but CT provides more detailed information. The combination of both methods would be ideal for developing and applying beak prostheses.

## Simple Summary

Birds with avulsion of their maxillary portion may have difficulty prehending food and could benefit from the placement of a prosthesis. For the proper development of a prosthesis, it is important to understand the characteristics of the beak that needs to be reconstructed. Thus, the present study evaluated the beaks of Neotropical birds— parakeets, red-legged seriemas, and black vultures—using CT scans, a dental intraoral scanner, and macroscopic analysis. Beak length, width, and height were measured on 3D images from CT and the dental scanner, while macroscopic measurements were also conducted. *Naris* dimensions were assessed on 3D scans, and the area of the beaks in cm^2^ was calculated in a sagittal view using a closed polygon tool. Some variations between imaging methods were observed, with CT providing more detailed data. The combination of both techniques is recommended for the development of beak prostheses.

## 1. Introduction

Birds use their beaks as a tool for numerous functions, such as feeding, defense, attack, object manipulation, social and sexual interaction, nest building, and locomotion, among others [1,2,3]. Partial beak loss can occur in any species, but birds with long or wide beaks are more prone to injury, including parrots, toucans, ducks, and hornbills [3,4]. Avulsion can be total or partial, with the latter involving the tip or a quadrant of the beak [5]. The tip of the beak is considered very sensitive; for example, cutting a goose’s beak causes an increase in blood pressure, heart rate, and tear flow [1].

Birds cannot survive the total loss of the mandible; however, some species can adapt to the total loss of the maxilla [5]. Avulsions involving less than the rostral third of the maxilla commonly resolve through the regrowth of the injured portion, but larger lesions present permanent deformity [6,7]. Waterfowl with avulsion of the maxillary portion may have difficulty prehending food, and, in the case of ramphastids and storks, self-feeding may be compromised [7,8]. Therefore, these birds would benefit from the placement of a prosthesis [7]. Birds with a wider beak base are also more favorable candidates for the placement of a prosthesis [4].

To create a prosthesis, an impression can be taken from the rhamphotheca using alginate or silicone, and then a mold can be made from plaster or resin or designed and built with a 3D printer [3,7,9]. Additionally, computed tomography (CT), magnetic resonance imaging, laser scanning, and photogrammetry can be used to generate 3D digital files which can be printed in materials such as polylactic acid (PLA), acrylonitrile butadiene styrene (ABS), titanium, or even polyetheretherketone (PEEK) [3,10,11].

However, for the proper development of a prosthesis, it is important to understand the characteristics of the beak to be reconstructed. In this sense, CT is considered a valuable tool in birds, as there is no interference due to the superimposition of images and the examination time is shorter compared to magnetic resonance imaging [3,8,12]. Additionally, CT is considered the imaging modality of choice for bone imaging and model production [3]. The limitations include the spatial resolution of the CT scanner used and the size of the bird [3,8].

Digital impressions obtained through intraoral scanners (devices for capturing direct optical impressions) have been increasingly used in human dentistry for various purposes, including as a tool to assist in diagnosis, treatment planning, prosthesis manufacturing, surgery, orthodontics, and implantology [13,14,15]. These devices project a light source, which can be a laser or structured light, on the object to be scanned [13].

Thus, the present study aimed to assess the beaks of Neotropical birds from three different families using two scanning techniques, CT and a dental intraoral scanner, along with macroscopic analysis. The goal was to identify anatomical structures, establish measurement patterns, and create three-dimensional models. The hypothesis was that there may be differences in beak measurements between imaging methods. To the best of the authors’ knowledge, the dental intraoral scanner has not yet been evaluated for generating STL (stereolithography) files of bird beaks for 3D printing.

## 2. Materials and Methods

### 2.1. Species Selection

Six specimens per family were selected, including parakeets (*Psittacara leucophthalmus*)—family Psittacidae; red-legged seriemas (*Cariama cristata*)—family Cariamidae; and black vultures (*Coragyps atratus*)—family Cathartidae. These were free-living adult birds found dead for reasons unrelated to the study. The cadavers were stored in a freezer at −20 °C until use. For imaging exams, the cadavers were thawed at room temperature. After macroscopic evaluation, one beak from each family was sectioned longitudinally for comparison with the imaging exams.

### 2.2. Computed Tomography

**Figure 1:**
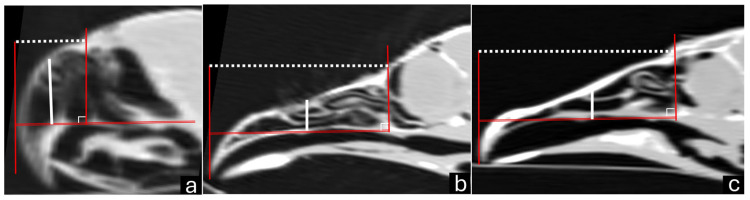
Sagittal computed tomography images of parakeet (**a**), red-legged seriema (**b**), and black vulture (**c**) beaks. Length measurement (white dotted line) from tip of the maxillary rostrum to anterior edge of skull (red lines). Height measurement on transverse axis in middle of *naris* (solid white line).

The CT scanning of the beak was performed with the bird in ventral recumbency. Transverse images were taken from the beak’s tip to its base using a spiral scanner (Shimadzu SCT-7800CT, Kyoto, Japan) with settings of 120 kVp, 1 mm slice thickness, and a pitch of 1.3 mm. Multiplanar reconstruction (MPR) was performed in the sagittal and dorsal planes. Finally, a three-dimensional (3D) reconstruction was created using the RadiAnt DICOM Viewer software, version 2024.2 (Medixant, Poznan, Poland). The upper beak was measured (Figure 1) on the sagittal view for the length along the longitudinal axis (from the tip of the maxillary rostrum to the anterior edge of the skull) and height on the transverse axis in the middle of the *naris*. The same measurements were performed on the 3D images.

**Figure 2:**
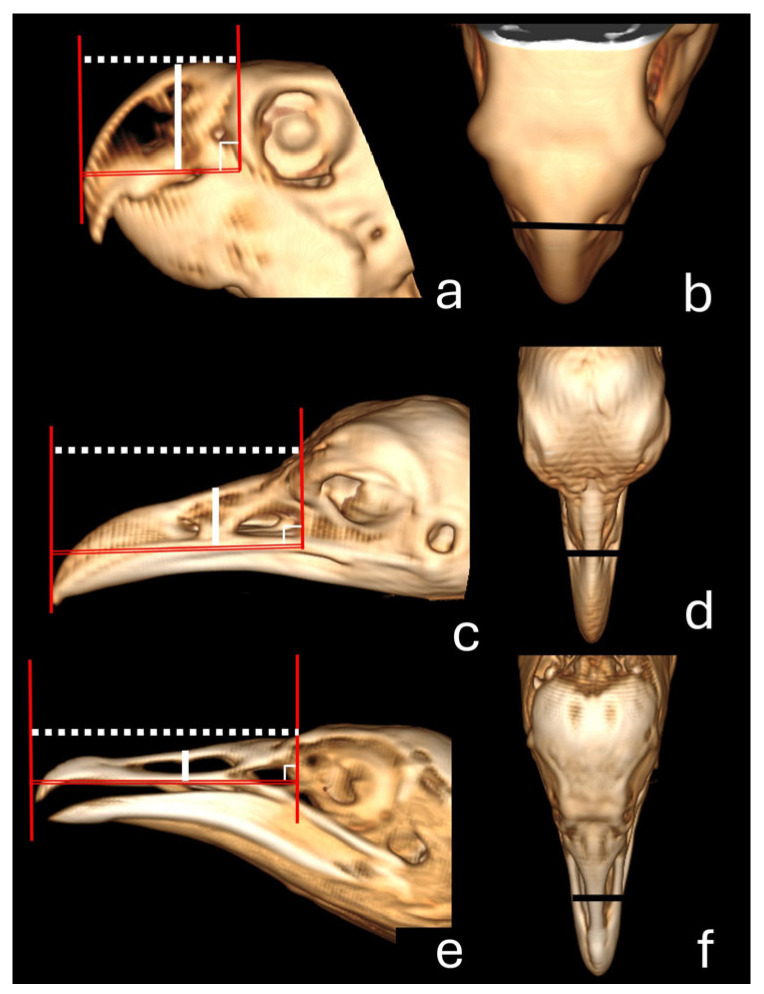
Three-dimensional tomographic images in the lateral (**a**,**c**,**e**) and dorsal (**b**,**d**,**f**) views of the beaks of parakeet (**a**,**b**), red-legged seriema (**c**,**d**), and black vulture (**e**,**f**). Observe the length (white dotted line) measured from the tip of the maxillary rostrum to the anterior edge of the skull (red lines) and height (solid white line) in the lateral view (**a**,**c**,**e**) and width (solid black line) in the dorsal view (**b**,**d**,**f**).

**Figure 3:**
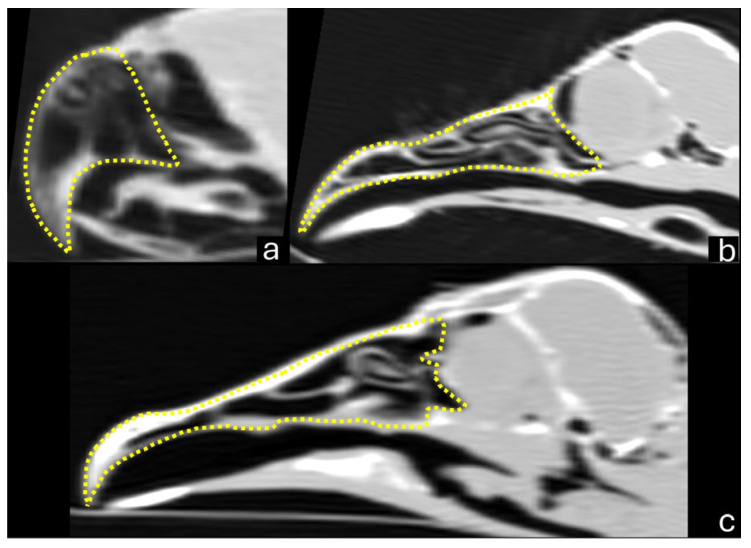
Sagittal computed tomography images (lung window) demonstrating the area measurement of the beak area (dotted yellow lines) for the parakeet (**a**), red-legged seriema (**b**), and black vulture (**c**).

In addition, the beak width was measured in the middle of the *naris* area in the 3D dorsal view, and the length and height of the *nares* were measured in the 3D lateral view (Figure 2). The beak was also outlined using the closed polygon tool in the lung window, generating an area in cm^2^ (Figure 3). A three-dimensional model was generated in STL (STereoLithography) format. All measurements and models were made using the RadiAnt DICOM Viewer software (Medixant, Poznan, Poland).

### 2.3. Dental Intraoral Scanner

**Figure 4:**
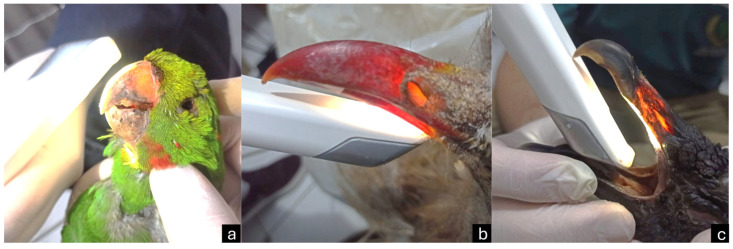
Scanning of parakeet (**a**), red-legged seriema (**b**), and black vulture (**c**) beaks with dental intraoral scanner.

**Figure 5:**
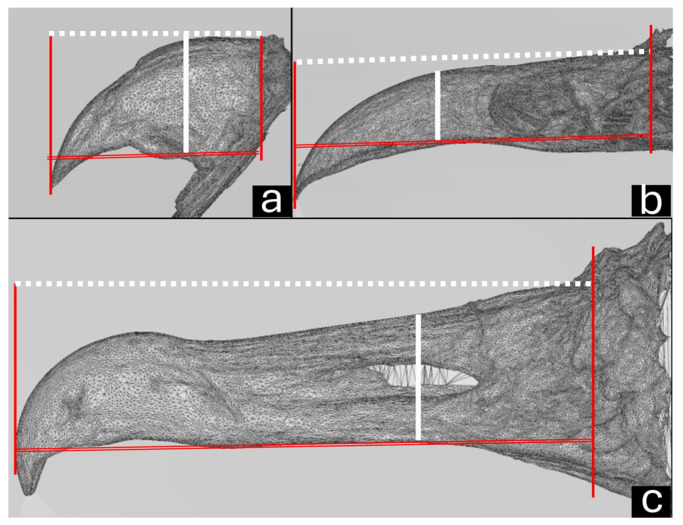
STL images in the lateral view of parakeet (**a**), red-legged seriema (**b**), and black vulture (**c**) beaks obtained with a dental intraoral scanner. Observe the length (white dotted line) measured from the tip of the maxillary rostrum to the anterior edge of the skull (red lines) and height (solid white line).

The beaks were scanned using a dental intraoral scanner (Trios 3 Classic; 3Shape; Copenhagen, Denmark). Scanning was performed from the base to the tip of the beak, covering both external and internal surfaces (Figure 4). The images were then reconstructed in 3D form using the 3D Builder (Microsoft Corporation; Redmond, WA, USA). Measurements (length, height, width), similar to those obtained from 3D-CT scans, were conducted using the 3D Builder measure tool (Figure 5).

### 2.4. Macroscopic Measurements

The length, height, and width of the upper beak were measured with a caliper using the same anatomical landmarks as those used in the 3D beak measurements.

### 2.5. Statistical Analysis

After performing the Kolmogorov–Smirnov normality test, a one-way ANOVA was conducted, followed by Bonferroni’s post hoc test to compare measurements taken using CT, the dental intraoral scanner, and the macroscopic method within each species. For the height of the red-legged seriema, a Friedman test followed by Dunn’s test was utilized. Additionally, ANOVA followed by Bonferroni’s post hoc test was applied to compare measurements across species using CT, the dental intraoral scanner, and the macroscopic method, except for 3D height, where the Kruskal–Wallis test followed by Dunn’s test was applied. The Kruskal–Wallis test was used to assess the area in cm^2^. The significance level adopted was *p* ≤ 0.05. The statistical analysis was performed using GraphPad Prism version 8.4.2 (GraphPad Software, Inc., San Diego, CA, USA).

## 3. Results

### 3.1. Macroscopy, CT, and Dental Intraoral Scanner

**Figure 6:**
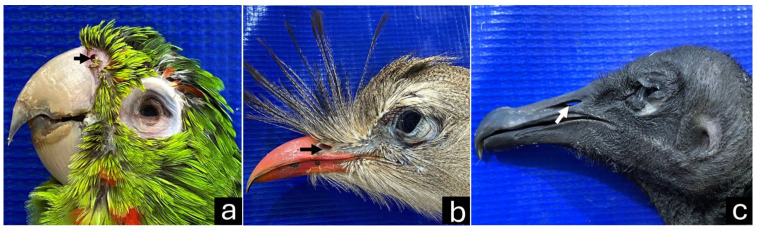
(**a**) A parakeet beak, with the *naris* located in the *cera* (black arrow). (**b**) A red-legged seriema beak, with the *naris* located laterally (white arrow). (**c**) A black vulture beak, with the perforated *naris* located in the middle third (white arrow).

Macroscopically, the parakeet beak was short with a hooked upper beak, and the rhamphotheca was light pinkish with some shades of gray. The *nares* were two round and distinct openings located in the *cera*, a waxy protuberance between the head and neck (Figure 6a). The red-legged seriema beak was relatively long and had a bright red color rhamphotheca. The upper beak was hooked, and feathers emerged from its base, forming a tuft. The *nares* were oblong and located laterally (Figure 6b). The black vulture beak was relatively long and had a hooked shape on the upper beak, and the rhamphotheca was mainly black. The tip of the upper beak was curved and lighter in color. The *nares* were located in the middle third of the beak, perforated and without a septum, and collimated laterally, allowing visibility through them (Figure 6c).

**Figure 7:**
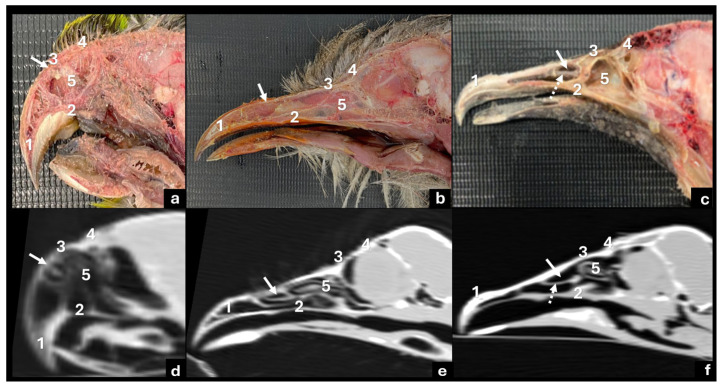
Macroscopic (**a**,**b**,**c**) and computed tomography images in sagittal view using lung window (**d**,**e**,**f**) of parakeet (**a**,**d**), red-legged seriema (**b**,**e**) and black vulture (**c**,**f**) beaks: 1—rostrum maxillare; 2—maxilla; 3—nasal bone; 4—frontal bone; 5—nasal cavity. White arrow shows *naris* topography and dotted arrow shows *naris* base.

In all the species, CT scans identified the external and internal structures of the beaks. The rostrum maxillare, maxilla, nasal bone, frontal bone, and nasal cavity were visualized in the sagittal plane (Figure 7). The parakeet also had its *cera* and the craniofacial hinge visualized (*zona flexoria craniofacialis*).

The CT measurements of the beaks are presented in Table 1.

**Figure 8:**
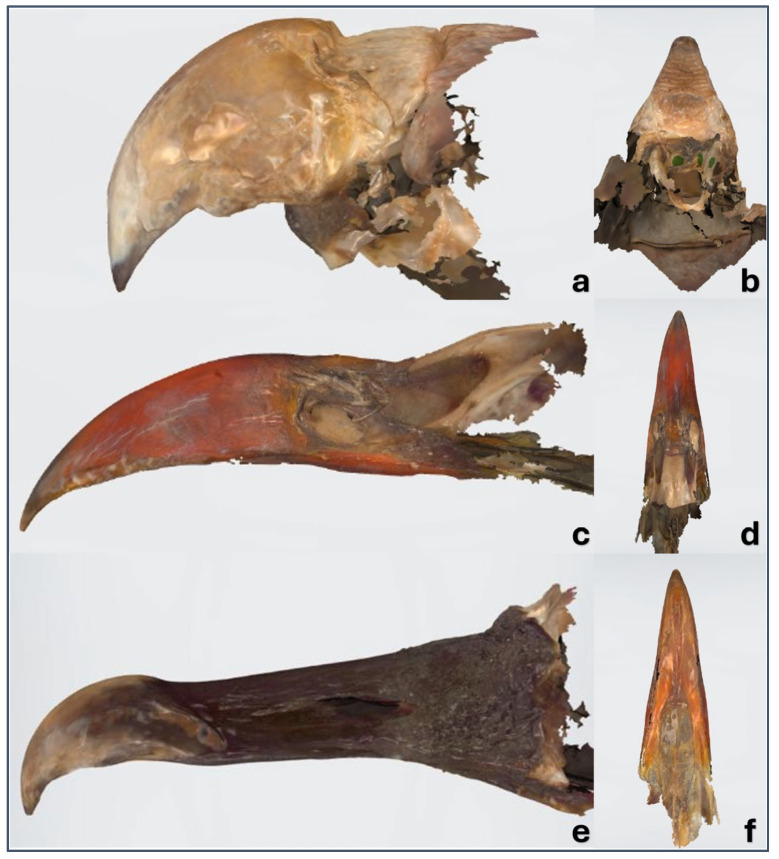
Images of parakeet (**a**,**b**), red-legged seriema (**c**,**d**), and black vulture (**e**,**f**) beaks obtained with dental intraoral scanner.

Scanning the beaks with a dental intraoral scanner took approximately five minutes. Powder opacification was not required to scan the beaks. Slender feathers that emerged from the beak were removed to avoid interference with scanning. The reproduction matched the original beak tones (Figure 8). The beak measurements from the 3D dental scanner, 3D-CT reconstruction, and macroscopy are presented in Table 2.

**Figure 9:**
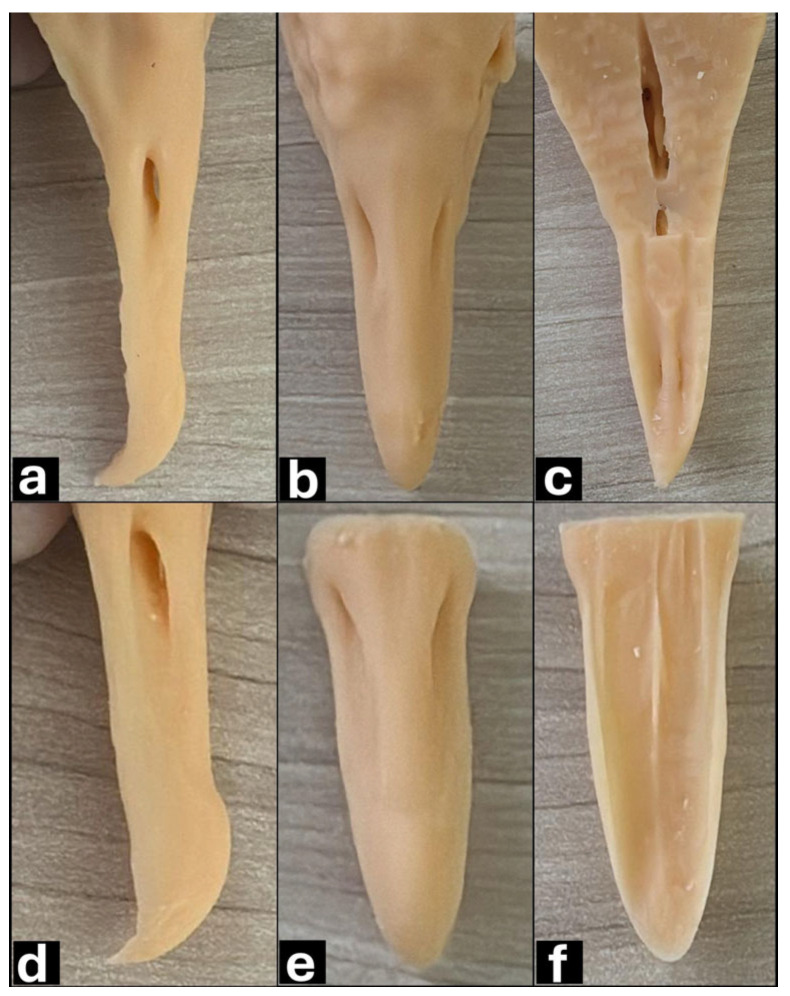
Rapid prototyping models in resin of black vulture beak generated from CT scan (**a**,**b**,**c**) and dental intraoral scanner (**d**,**e**,**f**). Lateral (**a**,**d**), dorsal (**b**,**e**), and (**c**,**f**) basal views.

Videos S1 and S2 (Supplementary Material) show the black vulture beak generated in STL using the CT and dental oral scans. Figure 9 shows an example of a rapid prototyping model in resin of a beak generated from a CT scan and a dental intraoral scanner.

### 3.2. Statistical Analysis

The comparison of beak measurements within each species showed that the dental intraoral scanner differed from both CT and macroscopic analysis in the height and width of the black vulture beaks, as well as the length and height of the parakeet beaks. The dental intraoral scanner also differed from CT in the length of black vulture beaks. The dental intraoral scanner, CT, and macroscopic analysis showed no differences in all measurements for the red-legged seriema beaks and in the width of the parakeet beaks. The statistical differences are presented in Table 3.

The height and length of the *nares* as well as the area (cm^2^) of the beaks differed among all species. The area and *naris* height were higher in the seriema beaks, followed by black vulture, and smaller in parakeet beaks.

## 4. Discussion

The hypothesis was confirmed as differences in beak measurements between the two imaging methods were verified. It is important to note that the three species selected for the study are commonly encountered in veterinary centers specializing in wildlife, particularly in Neotropical regions. The anatomical information obtained for each beak was used as a basis to evaluate the level of detail provided by the scanning methods. The CT scan provided detailed images of the anatomical structures of the beak, as observed macroscopically, highlighting the unique features of each species. In contrast, the dental intraoral scanner only captured external structures. Performing a CT scan on a living bird requires general anesthesia or deep sedation to prevent movement and stress, in addition to exposing the bird to ionizing radiation [16,17]. A beak scan can be performed with a dental intraoral scanner with the bird probably under physical restraint or light sedation. With an experienced operator, the scanning process is usually quick, as demonstrated in this study, where scanning took approximately five minutes for each bird’s beak.

The present study utilized a Trios 3 intraoral scanner, which employs the ultrafast optical sectioning technique established according to the confocal laser principle [14]. The accuracy of the dental scanner can be affected by several factors, including scanner technology (triangulation, confocal microscopy), material surface properties (reflective, matte, and porous), scanning software, light, ambient temperature, scanning strategy, and operator experience, among others [18,19,20]. The Trio 3 scanner has been demonstrated to have good accuracy in several studies [21,22]. Furthermore, the confocal plane is periodically changed at a certain frequency in this scanner, eliminating the need for the operator to adjust the scanner head to maintain a consistent distance from the object being scanned [14]. This feature can be advantageous when scanning the beak of a bird that is only under physical restraint.

The color of the rhamphothecas did not hinder the use of the dental scanner, and opacifying them with powder to create an opaque reflective coating before scanning was not necessary [13]. Additionally, the shape and color of the beaks were not affected during scanning, showing that the intraoral scanner used was sensitive enough to detect in-color impressions, which is an important aspect of the equipment’s ability [13]. However, testing on other beaks would be necessary, because digital impressions can be influenced by the surface of the object as different materials detect and reflect light differently [19].

Beak measurements obtained by CT and the dental intraoral scanner showed some statistical differences in parakeets and black vultures. The dental scanner must have both accuracy (agreement between the measurement result and a true value) and precision (agreement of values obtained by replicate measurements) [13]. These characteristics were observed mainly in the scanning of the seriemas’ beaks because there was statistical similarity among the three methods of measurement. The differences found in the other species may be related to the difficulty in determining the anatomical landmarks, especially the transition between the beak and skull, due to *naris* position and the presence of feathers in the *cera* region in parakeets. This demonstrates that, depending on the height of the lesion, CT is more sensitive than the dental intraoral scanner.

The functional morphology of the beak of each species must be taken into account when developing a prosthesis, considering the anatomical variation among species, as verified in the present study. For example, psittacines like parakeets have a short, curved upper beak with a wide base that fits over the lower beak [23]. They also have a special joint between the maxilla and the skull (craniofacial hinge) that gives them great mobility and strength in the beak [23,24]. On the other hand, members of the family Cathartidae, such as black vultures, have evolved specialized respiratory and olfactory systems for detecting and consuming carcasses [25]. They lack a septum between their *nares* and have narrow, elongated *nares* positioned farther back on a long, slender beak, allowing for the suction of soft material without obstruction [26,27]. The seriema has a strong hooked beak, often compared to the beak of a bird of prey, which is used to tear the flesh of its prey [28]. Thus, understanding these anatomical features is crucial for designing effective prostheses for birds.

In this regard, the CT scan enables reconstruction while preserving internal anatomical details, if necessary, whereas the intraoral scanner allows for the development of a solid structure. This consideration is crucial, especially when determining the position of the *nares* based on the bird’s trauma, so that models for larger and more destructive avulsions would be better developed with the aid of CT. On the other hand, the intraoral scanner provides greater surface precision compared to the CT scan, which should also be considered in the quality of the printed prosthesis.

Another key consideration is that birds with injuries in other areas of the body can also benefit from a CT scan. Additionally, from a practitioner’s perspective, if a CT scan has already been performed as part of the diagnostic process, the dental intraoral scan will only be used if the practitioner has the necessary equipment. It is important to note that several studies have successfully developed beak prostheses using solely CT scan images [29,30,31].

The details of beak thickness are important when fixing the prosthesis, for example, using screws. CT scans can help determine the optimal screw location and thickness, which is not possible with an intraoral scanner alone. Therefore, combining the capabilities of CT and an intraoral scanner would be more advantageous for virtually simulating the procedure before implementation compared to using either method independently.

The major limitation of this study was the use of only three bird species. Other species must be evaluated because the color and shape of the beak can influence the quality of dental intraoral scans. However, the findings from this study can be useful for future prosthetic research on bird beaks.

## 5. Conclusions

The two scanning techniques, CT and a dental intraoral scanner, enabled the assessment and creation of a three-dimensional model of the beaks of three Neotropical birds: parakeets, red-legged seriemas, and black vultures. Both techniques are effective, but CT provides more detailed information. The combination of both methods would be ideal for developing and applying a beak prosthesis.

## Figures and Tables

**Table 1:** Values of the measurements obtained by computed tomography of the upper beaks of the three Neotropical birds, including the length and height in sagittal view, sagittal area (cm^2^), and the length and height of the *nares* in three-dimensional view.

Bird/Number	Sagittal Length	Sagittal Height	Sagittal Area	3D *naris* Height	3D *naris* Length
Parakeet 1	2.13	1.33	2.35	0.1	0.1
Parakeet 2	2.18	1.35	2.5	0.12	0.1
Parakeet 3	2.0	1.36	2.34	0.12	0.1
Parakeet 4	2.32	1.28	2.5	0.1	0.11
Parakeet 5	2.06	1.3	2.3	0.1	0.1
Mean ± standard deviation	2.13 ± 0.12	1.32 ± 0.03	2.39 ± 0.09	0.11 ± 0.0	0.11 ± 0.01
Red-legged seriema 1	6.4	1.28	8.59	0.24	0.48
Red-legged seriema 2	6.69	1.25	7.13	0.25	0.46
Red-legged seriema 3	6.27	1.12	6.12	0.22	0.42
Red-legged seriema 4	7.21	1.32	8.32	0.2	0.45
Red-legged seriema 5	6.76	1.32	8.27	0.23	0.48
Mean ± standard deviation	6.66 ± 0.36	1.25 ± 0.08	7.68 ± 1.03	0.22 ± 0.01	0.45 ± 0.02
Black vulture 1	5.9	0.96	6.5	0.15	0.71
Black vulture 2	6.07	0.98	6.36	0.16	0.71
Black vulture 3	5.6	1.15	6.01	0.18	0.71
Black vulture 4	5.54	0.99	5.52	0.17	0.72
Black vulture 5	5.9	1.08	5.63	0.16	0.71
Mean ± standard deviation	5.80 ± 0.22	1.03 ± 0.08	6.0 ± 0.43	0.16 ± 0.01	0.71 ± 0.01

**Table 2:** Values of measurements obtained by three-dimensional computed tomography (CT), dental intraoral scanner, and macroscopy of upper beaks of three Neotropical birds.

Bird/Number	3D-CT Length	3D-Scanner Length	Macroscopic Length	3D-CT Height	3D-Scanner Height	Macroscopic Height	3D-CT Width	3D-Scanner Width	Macroscopic Width
Parakeet 1	2.09	1.65	2.23	1.36	1.51	1.31	1.43	1.35	1.4
Parakeet 2	2.11	1.54	2.15	1.4	1.41	1.38	1.33	1.22	1.36
Parakeet 3	2.01	2	2.29	1.37	1.58	1.35	1.4	1.44	1.41
Parakeet 4	2.15	2.1	2.17	1.29	1.41	1.3	1.44	1.52	1.42
Parakeet 5	2.05	1.76	2.13	1.3	1.42	1.3	1.45	1.55	1.43
Mean ± standard deviation	2.08 ± 0.05	1.81 ± 0.23	2.19 ± 0.06	1.34 ± 0.05	1.46 ± 0.07	1.33 ± 0.03	1.41 ± 0.05	1.41 ± 0.13	1.4 ± 0.03
Red-legged seriema 1	6.44	6.7	6.58	1.28	1.27	1.35	1.66	1.68	1.62
Red-legged seriema 2	6.62	7.3	6.68	1.27	1.3	1.37	1.62	1.69	1.64
Red-legged seriema 3	6.2	6.4	6.26	1.13	1	1.24	1.55	1.57	1.59
Red-legged seriema 4	7.21	7	7.09	1.31	1.8	1.3	1.69	1.65	1.65
Red-legged seriema 5	6.75	6.7	6.64	1.3	1.28	1.33	1.67	1.72	1.65
Mean ± standard deviation	6.65 ± 0.29	6.82 ± 0.34	6.64 ± 0.37	1.26 ± 0.07	1.33 ± 0.29	1.32 ± 0.05	1.64 ± 0.05	1.66 ± 0.06	1.63 ± 0.02
Black vulture 1	5.74	5.95	5.92	0.98	1.7	0.98	1.5	2	1.59
Black vulture 2	5.89	5.96	5.85	0.98	1.5	0.99	1.52	2	1.47
Black vulture 3	5.61	5.92	5.71	0.91	1	0.95	1.5	1.82	1.52
Black vulture 4	5.52	5.98	5.93	0.94	1.2	0.99	1.53	1.7	1.48
Black vulture 5	5.9	5.97	5.96	1.04	1.3	1.01	1.59	1.7	1.55
Mean ± standard deviation	5.73 ± 0.16	5.95 ± 0.02	5.87 ± 0.10	0.97 ± 0.04	1.34 ± 0.27	0.98 ± 0.02	1.53 ± 0.04	1.84 ± 0.15	1.52 ± 0.05

**Table 3:** Differences between measurements obtained by macroscopy, three-dimensional computed tomography, and dental intraoral scanner in black vulture and parakeet beaks.

Birds	Variables	*p* Values	Statistical Test
Parakeet	Length: 3D-CT × dental scanner; macroscopy × dental scanner	0.0070	Bonferroni
Parakeet	Height: 3D-CT × dental scanner; macroscopy × dental scanner	0.0024	Bonferroni
Black vulture	Length: 3D-CT × dental scanner	0.0244	Bonferroni
Black vulture	Height: 3D-CT × dental scanner; macroscopy × dental scanner;	0.0066	Bonferroni
Black vulture	Width: 3D-CT × dental scanner; macroscopy × dental scanner	0.0012	Bonferroni

## Data Availability

The data presented in this study are available on request from the corresponding author.

## Supplementary Materials

The following supporting information can be downloaded at: https://www.mdpi.com/article/10.3390/vetsci12040331/s1, Video S1: black-headed vulture beak generated in STL using the CT; Video S2: black-headed vulture beak generated in STL using a dental oral scan.

## Abbreviations

The following abbreviations are used in this manuscript:

CT	Computed tomography
3D	Three-dimensional
